# Supplementary material for: Urbanity and the dynamics of language shift in Galicia
Source: Nat Commun. 2019 Apr 11;10:1680. doi: 10.1038/s41467-019-09688-8 (PMC6459816; doi:10.1038/s41467-019-09688-8)
Supplement: Supplementary file 1 — Supplementary Information [file 41467_2019_9688_MOESM1_ESM.pdf]

Urbanity and the Dynamics of Language Shift in Galicia

Juane et al.

1.- Quantitative information for all the regions.

In Supplementary Table 1 we present all the relevant quantitative data corresponding to each of the regions considered for our analysis in the main text plus the averages over the four different groups (A to D). The parameter c was obtained by fitting the experimental data s described in the main text. The percentages of urban population for each region (u) was calculated directly from data available in [1, 2]. The values of *K* were estimated for a situation similar to Galicia. The growth factors were calculated as described in the Methods section considering all previous values. Additional details are in the caption.

Group	Region Number	Region Name	c	σ_c	$<c>$	$\sigma_{<c>}$	u	$<u>$	K	$<K>$	λ	$<\lambda>$
**A**	2	Ferrol, Eume y Ortegal	0,097	0,026	0,152	0,019	0,55	0,50	0,1110	0,1100	-0,25	-0,28
	5	A Coruña, Betanzos	0,183	0,051			0,63		0,1125		-0,27	
	12	Allariz, Maceda, Caldelas, Trives, Valdeorras	0,152	0,070			0,30		0,1059		-0,33	
	19	Pontevedra	0,143	0,054			0,51		0,1102		-0,28	
	20	Vigo	0,156	0,056			0,51		0,1101		-0,26	
**B**	6	A Barcala, Santiago, O Sar	0,38	0,17	0,338	0,018	0,53	0,43	0,1107	0,1087	-0,58	-0,62
	9	Lugo, A Ulloa, A Terra Chá, Meira	0,35	0,13			0,53		0,1106		-0,58	
	14	Ourense	0,149	0,039			0,73		0,1147		-0,72	
	16	A Parandanta, O Baixo Miño and O Condado	0,203	0,046			0,22		0,1043		-0,60	
	17	Caldas, O Salnés	0,40	0,17			0,26		0,1052		-0,58	
	18	O Morrazo	0,091	0,023			0,34		0,1068		-0,69	
**C**	4	Barbanza, Noia	0,66	0,34	0,592	0,097	0,29	0,28	0,1058	0,1056	-0,67	-0,63
	7	Chantada, Quiroga, Terra de Lemos	0,51	0,29			0,30		0,1059		-0,59	
	8	A Fonsagrada, Os Ancares, Sarria	0,65	0,36			0,20		0,1041		-0,69	
	10	As Mariñas	0,61	0,37			0,38		0,1076		-0,61	
	11	O Carballiño, O Ribeiro	0,34	0,12			0,23		0,1045		-0,59	
**D**	1	Arzúa, Ordes, Terra de Melide	0,78	0,46	0,73	0,20	0,15	0,18	0,1030	0,1036	-0,69	-0,70
	3	Bergantiños, Fisterra, Muros, Soneira, Xallas	0,70	0,51			0,13		0,1025		-0,78	
	13	A Limia Alta e Baixa, A Terra de Celanova, Verín, Viana	0,51	0,30			0,19		0,1039		-0,70	
	15	Deza-Tabeirós, Terra de Montes	0,85	0,47			0,25		0,1049		-0,63	

SupplementaryTable 1: Quantitative information for all the regions considered in the analysis presented in the main text [2]. Data is grouped according to the classification introduced in the main text and explicitly given in the **first** column. **Second** column is just a reference number and the **third** column presents the name of each region. **4th** and **5th** columns present the values of c as obtained from the experimental fits and the standard deviation (σ_c). **6th** column is the average of the c value for each group of regions ($<c>$) with the corresponding error in column **7th** ($\sigma_{<c>}$). **8th** column presents the fraction of urban population (u) calculated as the total urban population in that region over the total population in that region and column **9th** the averages corresponding to each group. **10th** column contains the K values used for the simulation of the Galician case. Column **11th** , the average of the K parameters for each region. Column **12th** is a list of all the growth factors calculated for each region (λ) and in column **13th ,** the corresponding averages for each group. The values of the c parameter are fits of the original data in [2].

2.- Definition of Singular Population Entities.

The definition of singular population entity according to the Spanish Instituto Nacional de Estadística (INE, Spanish Statistical Office) is “any inhabitable area of the municipality, inhabited or exceptionally uninhabited, clearly differentiated within it, and which is known by a specific denomination that identifies it without the possibility of confusion”.

The amount of 5000 inhabitants was chosen in this manuscript to discriminate between rural or urban settlements based on existing regulation. Locations with 5000 inhabitants or more, new regulations regarding construction and council representation apply. Also, direct inspection of the raw data shows a discontinuity in the series at precisely 5000 inhabitants (most likely due to the actual legislation).

3.- On the normalization of the variables (total population)

The model without the network term (Eqs. 1 in the main text) was theoretically analyzed and it was demonstrated that the total population was kept equal to one [3]. We did not perform a similar analysis (it is beyond the scope of this manuscript) when the network term is included (Eqs. 2 in the main text) but simple simulations for multiple sets of parameters demonstrate that the total population in each node is kept equal to 1. In Supplementary Fig. 1a we show the evolution of the variables (x_i, y_i, b_i) for an arbitrary region (node of the network). The total population is also plotted and the value is always equal to 1 as expected. Supplementary Fig. 1b shows the final values of the variables (x_i, y_i, b_i) at the stationary for all the nodes/regions in the network. Note that the total population is always equal to one.

The remainder of the subplots in Supplementary Fig. 1 show the same type of results for completely different values of the parameters (α, β). In all cases considered the total population is always equal to 1.

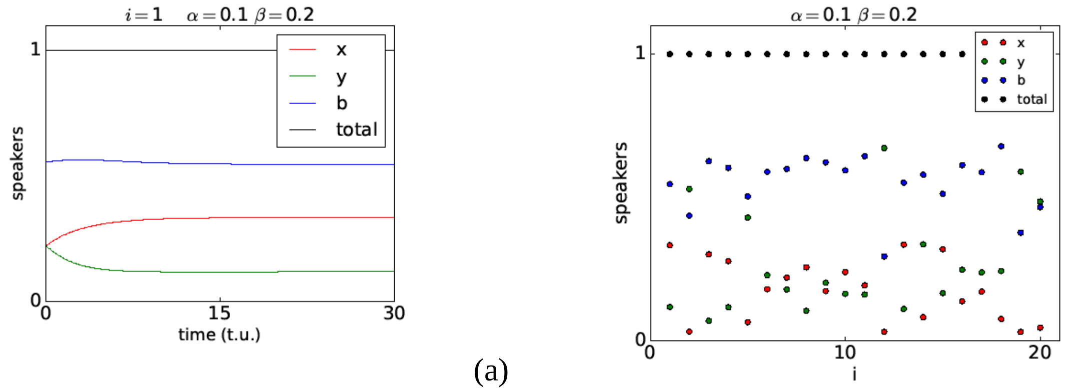

(a) (b)

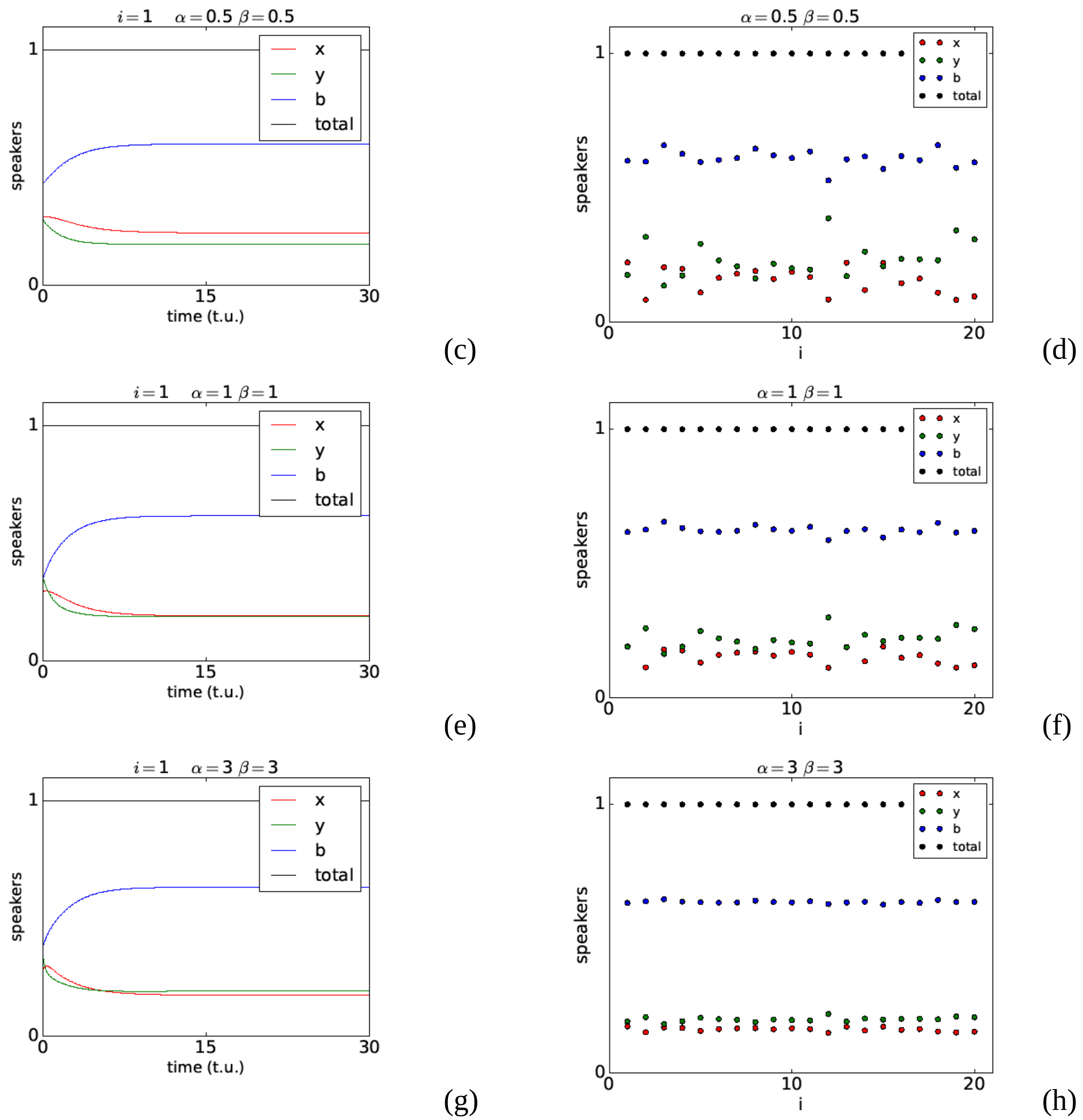

Supplementary Figure 1. Left column presents the temporal evolution of the model variables (x_i, y_i, b_i) in Eqs. 2 as well as the total population for an arbitrary node and different parameter (α, β) values. The right column presents the stationary values of the variables (x_i, y_i, b_i) and the total population for all the regions/nodes in the network. Each row corresponds with a different set of values for (α, β).

4.- Effect of the structure of K_i

All the results shown along the manuscript considered the simplest coupling between the nodes that actually accounts for the amount of urban population in each node. Here we analyze other, more complex, situations in order to provide evidence for the generality of the results.

Along the main manuscript, we analyze the temporal evolution of the speakers. As shown in the main text, the network term is given by

$$K_i \sum_{j=1}^{N} A_{ij}(x_j - x_i) \quad (1)$$

In the paper, the results were evaluated choosing the network weight factor as $K_i = \alpha(1 + \beta u_i)$ and the adjacency matrix elements as $A_{ij} = 1/N$. The initial case is set equal to $(x_{0,}y_0) = (0.3, 0.3)$ with a random perturbation of up to 10%. Note that the total population is maintained equal to 1 with the constraint $b = 1 - x - y$. Equations were integrated using Euler´s method with a time step $\Delta t = 10^{-4}$. The results are discussed along the main text.

a) Symmetric coupling. Another possible parameterization for the adjacency matrix could be

$$A_{ij} = \frac{1}{N}\frac{\alpha\left(1+\beta u_j\right)}{\sum_{j=1}^{N}\left(1+\beta u_j\right)} \text{ if } i \neq j$$

$$A_{ij} = 0 \text{ if } i = j. \quad (2)$$

In this case, as $K_i = \alpha(1 + \beta u_i)$ is still the same, the coupling coefficient becomes symmetric. It corresponds in a situation where a region feels each one of the other nodes of the network not only because of its amount of urban population but also because of the amount of urban population in the other nodes. It means that a very rural region will not feel the network at all, but those urban nodes will just feel those other nodes that are mostly urban.

The initial conditions and solving method used are as in the case discussed in the main text.

The results are in Supplementary Fig. 2. The plots on the left show the temporal evolution of the variables (x_i, y_i, b_i) of one single node of the network as well as the total population (that remains equal to 1 as expected). The plots on the right column, show the stationary state for each one of the 20 regions/nodes in the network with this configuration. Each row corresponds to a different set of the coupling parameters (α, β). Note that the total population still remains normalized to unity.

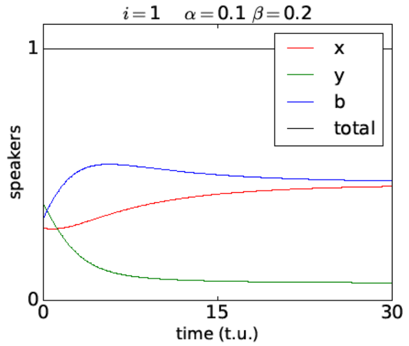

i = 1 α = 0.1 β = 0.2
x
y
b
total
speakers
time (t.u.)

(a)

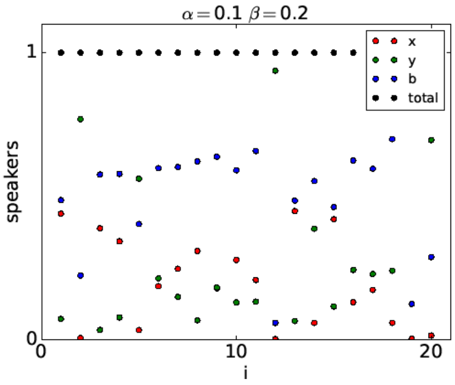

α = 0.1 β = 0.2
x
y
b
total
speakers
i

(b)

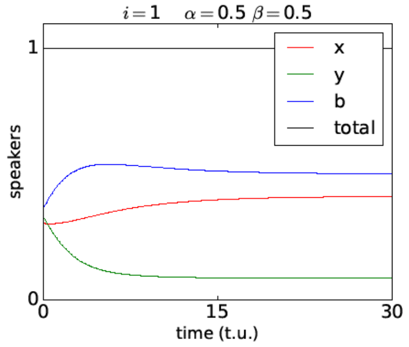

i = 1 α = 0.5 β = 0.5
x
y
b
total
speakers
time (t.u.)

(c)

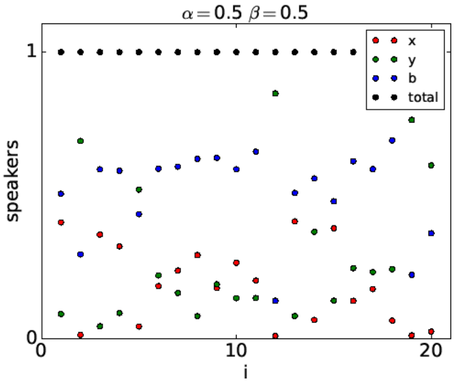

α = 0.5 β = 0.5
x
y
b
total
speakers
i

(d)

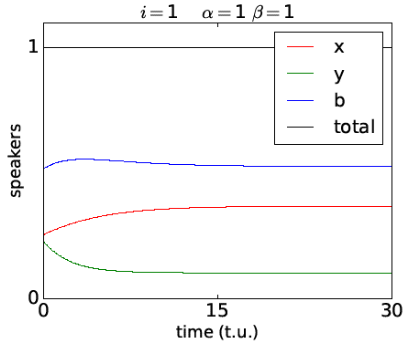

i = 1 α = 1 β = 1
x
y
b
total
speakers
time (t.u.)

(e)

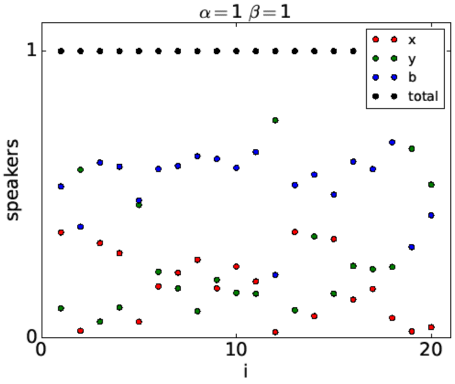

α = 1 β = 1
x
y
b
total
speakers
i

(f)

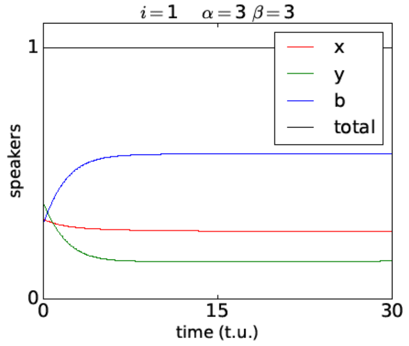

i = 1 α = 3 β = 3
x
y
b
total
speakers
time (t.u.)

(g)

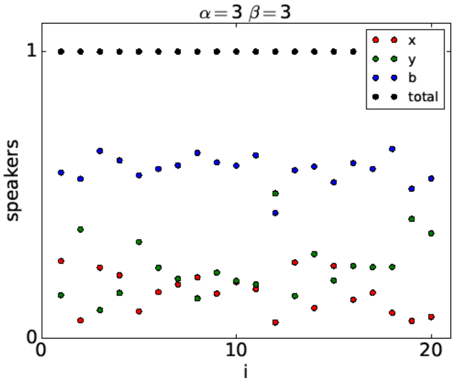

α = 3 β = 3
x
y
b
total
speakers
i

(h)

Supplementary Figure 2. Symmetric coupling coefficient. The left column presents the temporal evolution of the model variables (x_i, y_i, b_i) in Eqs. 2 (in the main text) with the symmetric coupling considered in this subsection as well as the total population for an arbitrary node and different parameter (α, β) values. The right column presents the stationary values of the variables (x_i, y_i, b_i) and the total population for all the regions/nodes in the network. Each row corresponds with a different set of values for (α, β).

Supplementary Fig. 3 plots the growth factors (λ_i) versus the coupling coefficient (K_i) for different values of the (α, β) parameters. Note that the growth factors for the Group A regions (more urban regions) are smaller (in absolute value) than those mostly rural regions (Group D). For this particular type of coupling, we did not find the reverse situation. This can be understood analyzing the coupling coefficients as the factor $(u_i \cdot u_j)$ strongly connects urban regions and dismisses connections between rural zones. Information is more accessible for urban zones.

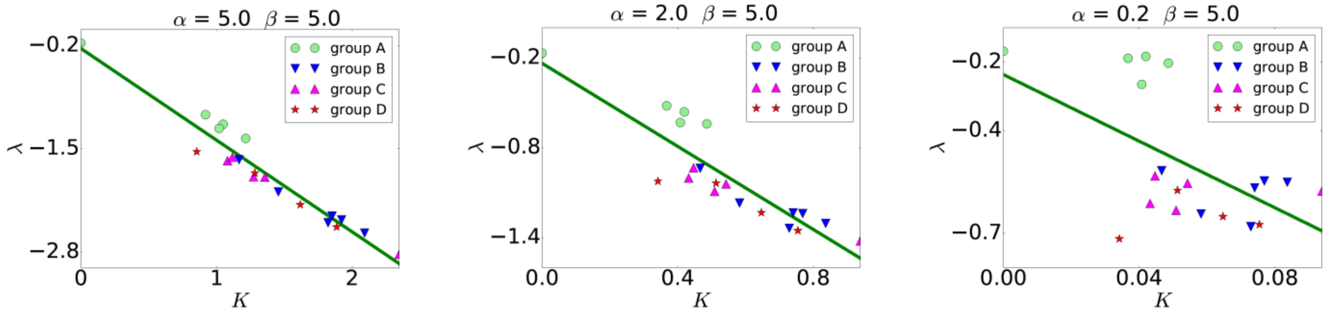

(a) (b) (c)

Supplementary Figure 3. Symmetric coupling coefficient. Growth factors (λ_i) versus the coupling coefficient (K_i) for different values of the (α, β) parameters.

b) Non-symmetric coupling coefficient weighted by the total urban population in each node. Another option for the adjacency matrix is given by

$$A_{ij} = \frac{1}{N} \frac{u_j p_j}{\sum_{j=1}^{N} u_j p_j} \qquad (3)$$

where u_j is the percentage of urban population in node j and p_j is the absolute value of the population in node j. thus $(u_j \cdot p_j)$ is the total urban population in node j. In this case, the contribution of each node is weighted by the total urban population in such a way that regions with low population play a less significant role than those with large populations (that in our case usually corresponds with predominantly Spanish-speaking regions).

Supplementary Fig. 4 plots on the right the temporal evolution of the variables (x_i, y_i, b_i) of one single node of the network as well as the total population (that remains equal to one as expected).

The plots on the right side, show the stationary state for each one of the 20 regions/nodes in the network with this configuration. Each row corresponds to a different set of the coupling parameters (α, β).

Supplementary Fig. 5 plots the growth factors (λ_i) versus the coupling coefficient (K_i) for different values of the (α, β) parameters. Note that the growth factors for the Group A regions (more urban regions) are smaller (in absolute value) than those mostly rural regions (Group D). Again, for this particular coupling, we did not find the reverse situation.

This parameterization strongly enhances inequality in the system suppressing the equalizer effect of the α parameter. Rural zones are excluded from the network as their urban population $u_j p_j$ is low. The information available comes from urban zones. As, in Galicia, urban is linked to Spanish and rural to Galician, urban zones become more connected due to the factor $u_j p_j$ that enables connections between urbans. Network effects, on the contrary, are depressed in rural zones.

The way that regions access information matters. The adjacency matrix used in the main paper allows egalitarian access to the information which is closer to the situation described in this paper.

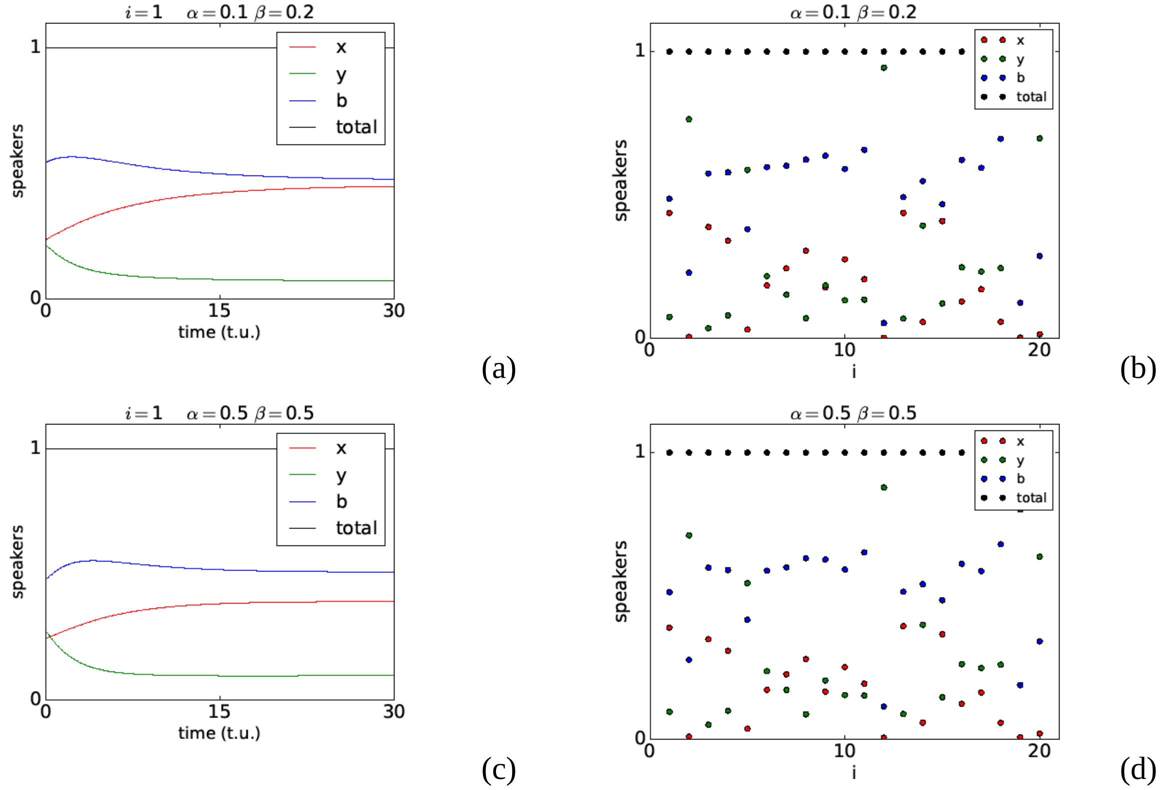

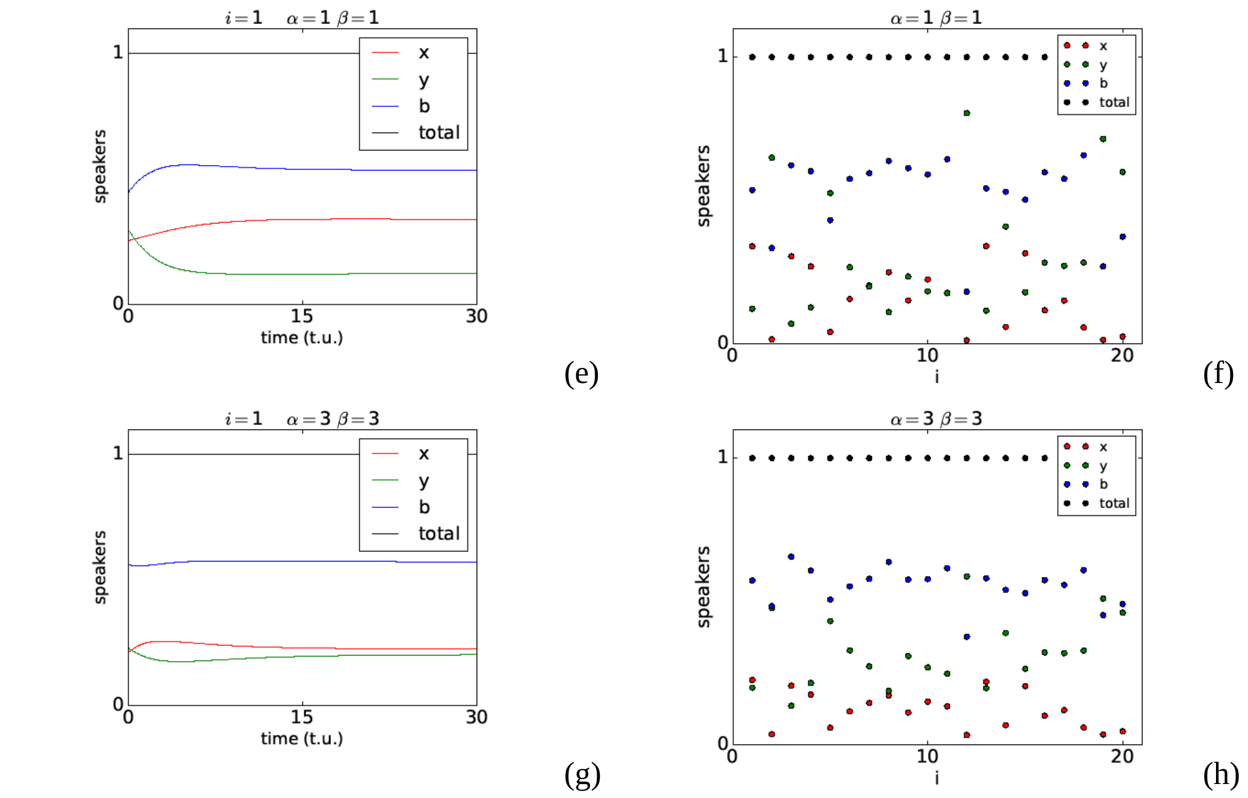

Supplementary Figure 4. Non-symmetric coupling coefficient weighted by the total urban population in each node. Left column presents the temporal evolution of the model variables (x_i, y_i, b_i) in Eqs. 2 (in the main text) with the non-symmetric coupling considered in this subsection as well as the total population for an arbitrary node and different parameter (α, β) values. The right column presents the stationary values of the variables (x_i, y_i, b_i) and the total population for all the regions/nodes in the network. Each row corresponds with a different set of values for (α, β).

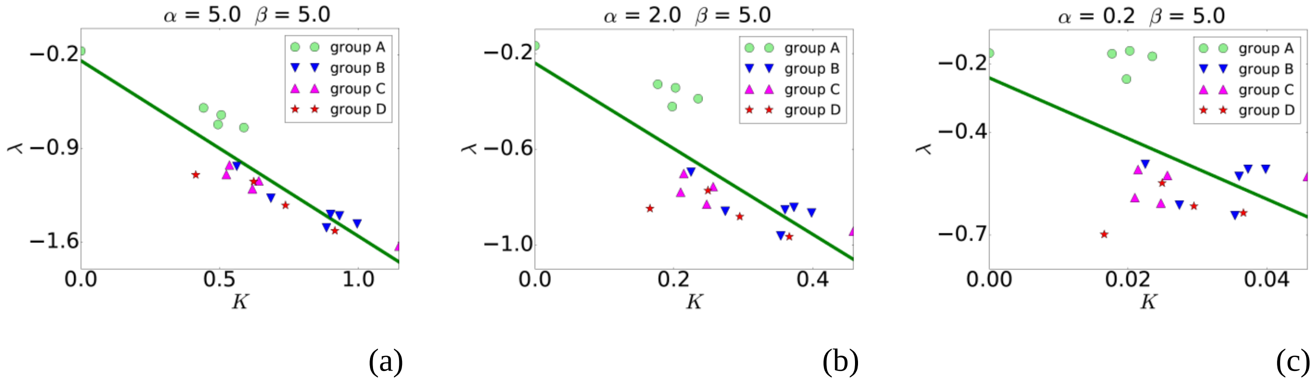

Supplementary Figure 5. Non-symmetric coupling coefficient weighted by the total urban population in each node. Growth factors (λ_i) versus the coupling coefficient (K_i) for different values of the (α, β) parameters.

References

[1] Instituto Nacional de Estadística (Spanish Statistical Office), http://www.ine.es. Instituto Galego de Estatística (Galician Statistical Office), http://ige.eu.

[2] Instituto Galego de Estatística (Galician Statistical Office) https://www.ige.eu/web/mostrar_actividade_estatistica.jsp?idioma=gl&codigo=0206004

[3] Otero-Espinar, M.V., Seoane, L.F., Nieto, J.J. & Mira, J. An analytic solution of a model of language competition with bilingualism and interlinguistic similarity. Physica D 264, 17-26 (2013).
